# Automated linac downtime tracking across 4 radiation oncology clinics within a single hospital system utilizing statistical process control

**DOI:** 10.1002/acm2.70863

**Published:** 2026-10-14

**Authors:** Caroline E. Serafini, Matthew Thomas, Xiaofeng Yang, Pretesh Patel, Justin Roper, Maxine Washington, Anees Dhabaan, Hania Al‐Hallaq

**Affiliations:** ^1^ Department of Radiation Oncology Winship Cancer Institute, Emory University Atlanta Georgia USA

**Keywords:** linear accelerator, downtime surveillance, statistical process control

## Abstract

**Background:**

Linear accelerator (linac) downtime disrupts clinical workflow and may delay patient treatment; however, downtime events are frequently undocumented or recorded in informal reporting systems. Data‐driven approaches using routinely collected operational metrics may improve downtime surveillance and provide insight into system‐level adaptation.

**Purpose:**

This study aims to evaluate if statistical process control (SPC) methods can be applied to routine treatment and scheduling data from an oncology information system (OIS) to identify linac downtime events and characterize operational response across multiple radiation oncology clinics within a single hospital system.

**Methods:**

Daily machine‐level operational data from 10 linacs across 4 clinics were analyzed over 29 months (January 2024–May 2026). Machine metrics included daily patient volume, cancellations, and operating hours. For each machine, rolling 45‐day baseline means and standard deviations were computed, and SPC rules (± 3σ control limits and > 10 cancellations / day) were applied to detect downtime days. SPC‐flagged events were compared with email‐based downtime event logs to calculate sensitivity, specificity, positive predictive value (PPV), and negative predictive value (NPV). Downtime events were categorized by cause, and intra‐hospital patient redistribution was assessed by identifying concurrent > 4σ volume increases on alternate machines.

**Results:**

Over the 29‐month study period, 138 downtime events were documented in downtime event logs. SPC identified 23 additional events exhibiting operational signatures consistent with downtime. Using reported events as the reference standard, SPC achieved sensitivity 0.70, specificity 0.99, PPV 0.79, and NPV 0.99. Downtime frequently resulted in compensatory increases in patient volume on alternate machines, with 41 transfer pairs identified across three hospitals. When the reported downtime cause was specified, machine mechanical causes accounted for the largest proportion (48%) of downtime events.

**Conclusions:**

SPC applied to routinely collected operational data provides a scalable framework for automated linac downtime detection. Integration of automated monitoring with structured reporting may improve downtime surveillance and inform proactive operational planning in radiation oncology

## 1 INTRODUCTION

Linear accelerators (linacs) are central to the delivery of modern radiation therapy, supporting increasingly complex treatment techniques including intensity‐modulated radiotherapy (IMRT), volumetric modulated arc therapy (VMAT), and stereotactic body radiotherapy (SBRT). As fractionation schedules shorten and adaptive workflows grow, uninterrupted machine availability becomes essential to maintaining treatment continuity and preventing detrimental delays that may compromise tumor control or necessitate compensatory adjustments to treatment plans.^1^, ^2^, ^3^, ^4^

Consistent with quality management frameworks that emphasize proactive identification and monitoring of risks to treatment delivery, machine reliability and operational resilience represent important components of radiotherapy quality.^5^, ^6^, ^7^, ^8^ However, downtime reporting often relies on manual processes, such as e‐mail reporting and informal communication, which may incompletely capture disruptions, particularly brief events or those resolved without escalation.^9^, ^10^ Consequently, systematic approaches for identifying downtime events and longitudinally monitoring linac performance using routinely collected operational data remain limited, highlighting the need for automated surveillance systems capable of assessing machine performance relative to established process control limits.

Statistical process control (SPC), originally developed for industrial quality assurance, provides a structured method for distinguishing routine variation from significant deviations in time‐series data.^11^, ^12^, ^13^, ^14^, ^15^, ^16^ While SPC has been applied in radiation oncology for equipment performance, dosimetric quality assurance, and workflow monitoring, its application to operational scheduling data for the purpose of downtime detection represents a novel extension of methodology.^17^, ^18^, ^19^, ^20^, ^21^ Compared with fixed‐threshold approaches, SPC adapts to institutional baseline variation and seasonal scheduling fluctuations, making it particularly well‐suited for operational metrics that evolve over time such as quantifying linac downtime.

This study evaluates whether SPC applied to routinely collected treatment and scheduling data could serve as a practical framework for automated identification of linac downtime events across a multi‐clinic hospital system, including characterization of intra‐hospital patient redistribution patterns during machine outages. Because the approach relies exclusively on data routinely collected within standard oncology information systems, it has the potential to be implemented with minimal additional infrastructure and automated through scripted analysis. By integrating daily operational metrics with manually reported downtime event logs over a 29‐month period, the diagnostic performance of SPC‐based inference was evaluated against documented downtime occurrences. In addition, patterns of patient redistribution associated with machine outages are characterized to establish a scalable, data‐driven framework for downtime surveillance that may support quality management initiatives, benchmarking of machine reliability, and evidence‐based operational planning in radiation oncology.

## 2 METHODS

### 2.1 Study setting and data sources

This retrospective study included 10 linear accelerators distributed across four radiation oncology clinics within a single hospital system. The linac fleet consists of a mixed‐vendor configuration of Varian and Elekta systems, with machine ages ranging from 3 to 15 years at the time of data collection. All four clinics operate within a shared oncology information system (OIS) (ARIA, Varian Medical Systems), providing a unified data environment for retrospective extraction across sites. The hospitals are part of the same health system network, with a centralized IT infrastructure.

Data were collected over a 29‐month period from January 1, 2024 through May 31, 2026. Machine schedule and treatment‐record data were obtained from the ARIA database. Schedule records were analyzed to classify appointment outcomes (completed, canceled, etc.), and treatment‐delivery records were used to evaluate machine utilization metrics, including beam‐on time and treatment‐day duration. The clinical environment across the four sites encompasses a range of treatment modalities including volumetric modulated. arc therapy (VMAT), stereotactic body radiotherapy (SBRT), stereotactic radiosurgery (SRS), total body irradiation (TBI), and total skin electron therapy (TSET). The number of patients treated on each machine ranged from 10–40 (see Figure 3 and Appendix Section S.C).

### 2.2 Operational metrics and downtime definition

Machine downtime was defined as any period during which a linac was unable to deliver scheduled patient treatments due to technical, software, mechanical, or facility‐level causes.

For each machine and each calendar day, three operational metrics were utilized from the treatment database: (1) the total number of patients treated (daily volume), (2) the number of canceled treatment appointments, and (3) the total operating hours of the machine from the first to last treated patient. Daily summaries were generated per machine. Scheduled non‐treatment days (e.g., weekends and holidays) were excluded from analysis.

Specifically, appointment classifications (i.e., completed vs. canceled) and daily operating durations (i.e., time of first to last treated patient) were extracted directly from ARIA treatment records using scripting. Cancellation rationale was not available as a structured field in the OIS and was instead classified manually from institutional email‐based event logs, as described below.

Downtime events recorded in institutional event logs were considered “reported downtime” or the ground truth for comparison. These included both machine‐specific events and institution‐wide cancellations (e.g., weather or system‐wide IT outages).

### 2.3 Statistical process control framework

To detect anomalous operational patterns consistent with downtime, statistical process control (SPC) methods were applied independently to each machine and each metric. Specifically, Shewhart‐style individuals control charts were constructed for each metric, using a rolling rather than a fixed mean to allow control limits to scale with scheduling fluctuations and gradual long‐term operational shifts inherent in a busy clinical environment.^11^ The selection of rolling window length and control limit threshold was guided by empirical evaluation of alternative parameter combinations, summarized in Appendix Sections S.A–S.B. Briefly, rolling window lengths shorter than 30 days reduced specificity due to instability in baseline estimation, while windows longer than 60 days reduced sensitivity to sustained operational shifts. For this dataset, a 45‐day window was identified to provide the best balance between sensitivity and specificity. Control limit evaluation demonstrated that ± 3σ minimized overall classification error relative to more permissive thresholds, consistent with conventional SPC practice for distinguishing special‐cause from common‐cause variation.

For each machine, rolling 45‐day baseline statistics were computed for the mean (μ) and standard deviation (σ). The rolling window excluded the current day to avoid contamination of baseline estimates. Control limits were defined as the Upper Control Limit (UCL) = μ + 3σ and the Lower Control Limit (LCL) = μ ‐ 3σ.

SPC inference rules were applied to flag potential downtime events. A day was classified as SPC‐positive if any of the following occurred: (1) daily patient volume fell below the LCL, (2) daily operating hours fell below the LCL, (3) daily cancellations exceeded the UCL, or (4) total cancellations exceeded 10 patients in a single day

This approach enabled the identification of downtime through the combined assessment of reductions in throughput and spikes in cancellations. The rolling window length and control limit threshold were selected based on empirical evaluation of classification performance across alternative parameter settings (Appendix Sections S.A—S.B). Days flagged by SPC without a corresponding event log entry were classified as SPC‐inferred downtime. This process is outlined in Figure 1.

**FIGURE 1:**
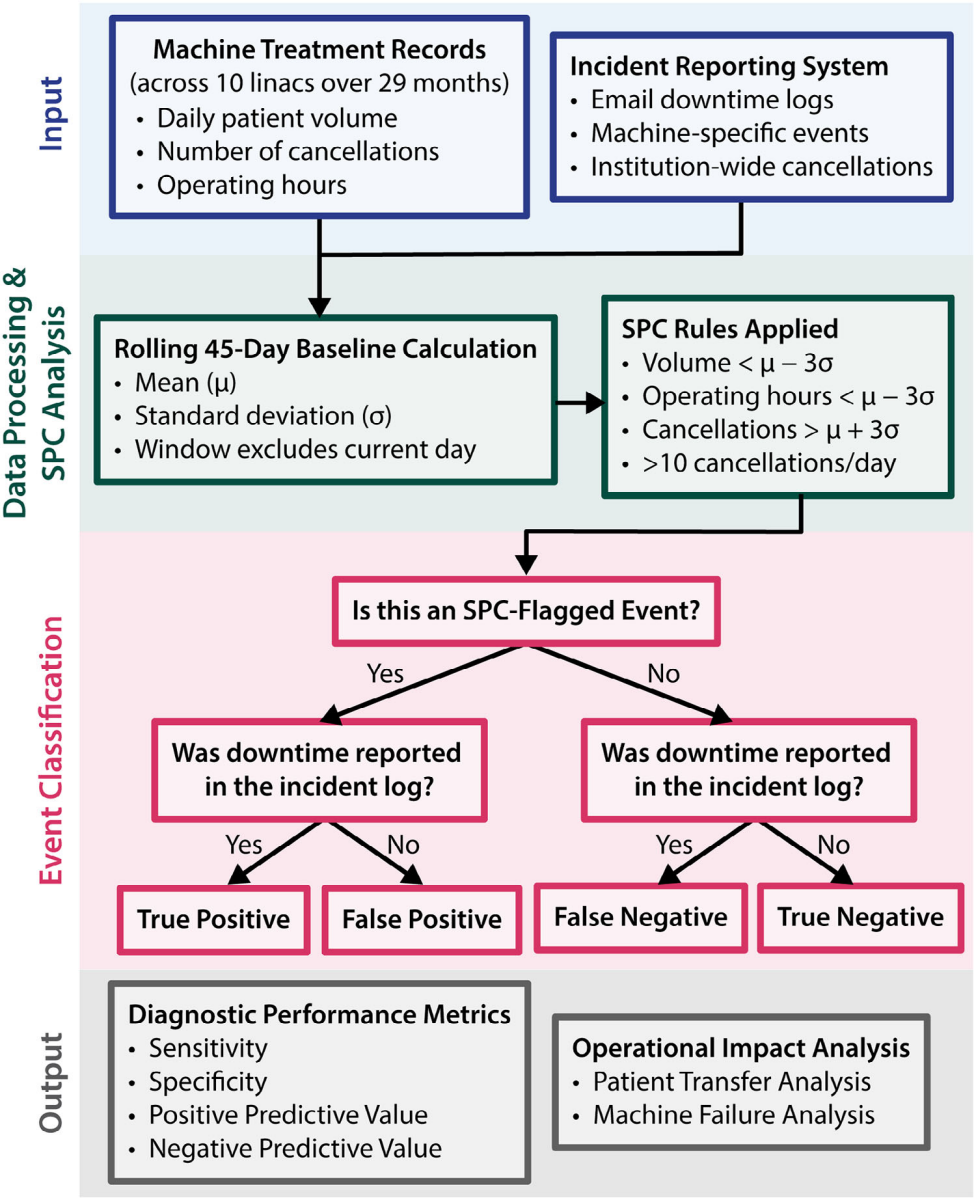
Workflow for automated detection and evaluation of linac downtime events using SPC applied to daily machine‐level operational data from the record‐and‐verify system.

### 2.4 Performance evaluation

The performance of the SPC‐based detection framework was evaluated using event log–reported downtime as the reference standard. For the first 18 months of the study, events were reported via internal e‐mails. For the final 11 months of the study period, departmental emphasis on downtime reporting was increased, accompanied by the introduction of a structured internal reporting form. Notably, the improved reporting system standardized event reporting capturing the severity of event, including brief or clinically minor interruptions documented by therapists (e.g., events noted as “down 15 min” or “maintenance needed”) that may not have been captured or formally escalated under the prior system. During this period, an emphasis was placed on educating staff to utilize the form to capture downtime events of any duration.

For each machine, the sensitivity, specificity, positive predictive value (PPV), and negative predictive value (NPV) were calculated. True positive events were defined as days both reported in event logs and flagged by SPC. False positives were SPC‐flagged days not present in event logs. False negatives were reported downtime events not identified by SPC.

### 2.5 Intra‐hospital patient redistribution analysis

To evaluate operational adaptation during downtime events, intra‐hospital patient transfer patterns were assessed. For each hospital, days with confirmed or SPC‐inferred downtime on one machine were examined for concurrent increases in patient volume on other machines within the same clinic. A redistribution event was recorded when downtime on a single machine coincided with a > 4σ increase in daily patient volume on another machine within the same hospital. Transfer pairs were tabulated to characterize patterns of compensatory scheduling across machines.

### 2.6 Downtime event classification

Downtime events were categorized into four groups: mechanical, weather‐related, information technology (IT)‐related, and operational/other (unspecified or workflow‐related). Mechanical events included hardware failures (i.e., MLC faults, beam generation components, imaging systems, gantry and couch issues) of the treatment machine. Events attributed to weather‐related factors or IT‐related issues were classified separately. All other events were categorized as operational and other. These consisted of non‐machine‐related stoppages such as medical emergencies, treatment room issues such as a door failure, or sanitary events as well as events without a clearly documented root cause.

## 3 RESULTS

### 3.1 Downtime event summary

Over the 29‐month study period (January 2024–May 2026), 138 downtime events were documented in the institutional event logs across 10 linacs at four hospitals. Machine‐specific interruptions accounted for 66 events, 40 were institution‐wide cancellations (30 weather‐related events and 10 system‐level IT disruptions) occurring on four calendar dates, 7 were IT‐related issues affecting individual machines, and the remaining 25 were operational events impacting individual machines, as seen in Figure 2. Here, mechanical events correspond to the largest proportion of downtime events (48%) across all clinics and machines. Across all machines, downtime events were not uniformly distributed, with some machines exhibiting more downtime events than others, as seen in Figure 2 and Table 1.

**FIGURE 2:**
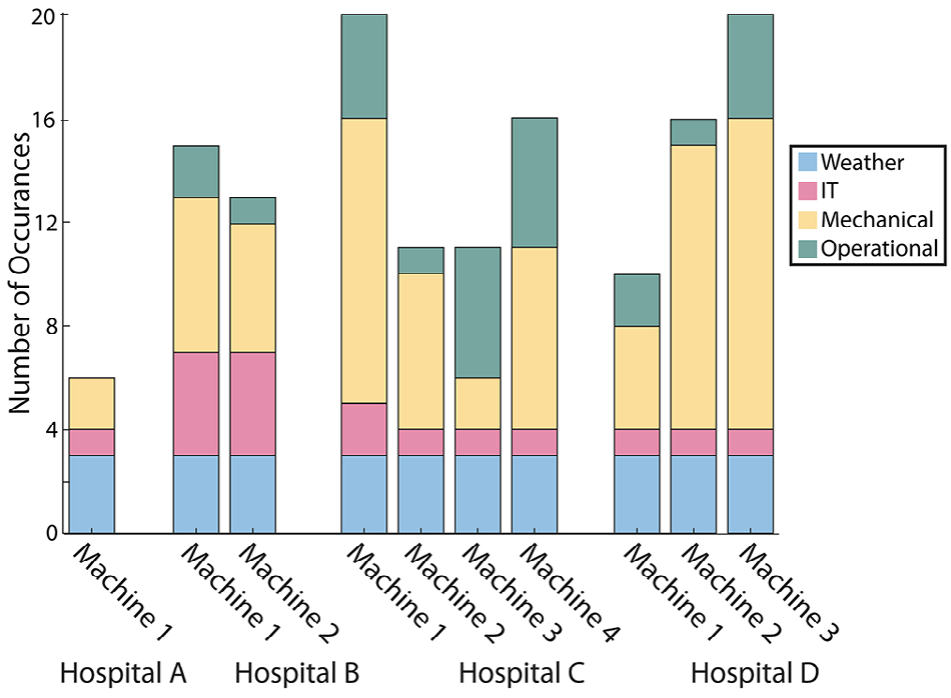
Distribution of reported linac downtime events categorized by general cause across all hospitals over 29 months.

**TABLE 1:** Classification of linac downtime events over the full 29‐month study period. Events are categorized as true positives (email‐reported and SPC‐predicted), false positives (SPC‐predicted without corresponding email documentation), and false negatives (email‐reported but not identified by SPC), stratified by hospital and machine.

		True Positive (Email Reported & SPC‐Predicted)	False Positive (SPC‐Predicted & Not Email Reported)	False Negative (Email Reported & Not Predicted by SPC)
**Hospital A**	Machine 1	6	3	0
**Hospital B**	Machine 1	12	2	6
	Machine 2	16	1	4
**Hospital C**	Machine 1	16	4	11
	Machine 2	15	2	3
	Machine 3	8	4	5
	Machine 4	8	1	2
**Hospital D**	Machine 1	14	1	4
	Machine 2	15	2	12
	Machine 3	18	3	7

### 3.2 SPC‐based downtime detection performance

Application of SPC criteria identified 23 days with irregular operational metrics that were not recorded in event logs, representing potential underreported downtime events. These events were identified through deviations in one or more monitored metrics, including decreases in patient volume and operating hours, as well as increases in cancellations beyond defined control limits.

In several cases, SPC flagged multiple machines concurrently, producing patterns consistent with institution‐ or clinic‐wide disruption. Examples of these deviations are seen by the darker red data points in Figure 3 and Appendix Sections S.C—S.E.

**FIGURE 3:**
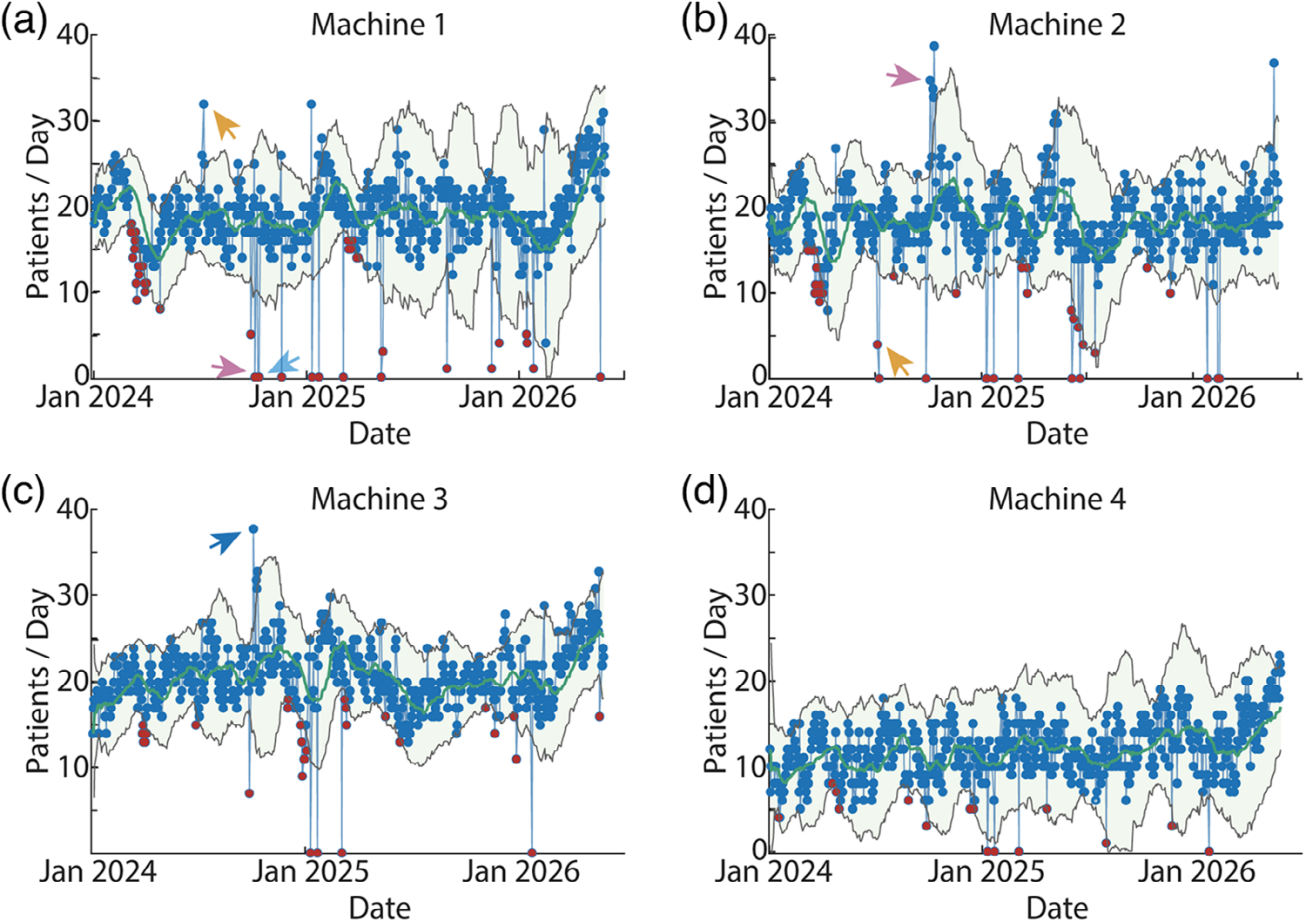
Representative statistical process control (SPC) charts for four linacs at Hospital C over the 29‐month study period. Daily patient volume is shown with rolling 45‐day baseline means (solid line) and ± 3σ control limits (shaded region). Red markers indicate reported downtime events, while additional SPC‐flagged events without corresponding documentation are visible as control limit violations. Arrows highlight paired patient transfers within the radiation oncology clinic.

Using event log–reported downtime as the reference standard, the SPC framework achieved a sensitivity of 0.70, specificity of 0.99, PPV of 0.84, and NPV of 0.99 when evaluated against event log–reported downtime as the reference standard over the full 29‐month study period. The number of events identified by SPC per machine are shown in Table 1.

Representative SPC control charts for patient volume at four machines at a single hospital are shown in Figure 3. Downtime events are identified as those that fall outside the normal‐process variation (shaded region). Distinct clusters indicating operational variations, which can indicate recurrent issues on a single machine, can be seen. SPC charts for all downtime variables for all machines can be seen in Appendix Sections S.C—S.E.

### 3.3 Intra‐hospital patient redistribution

Of the downtime events across the three hospitals with multiple machines capable of accepting patient transfers, 41 transfer pairs were identified, representing measurable compensatory increases in volume on alternate linacs. Several examples of these transfer days are indicated by the colored arrows in Figure 3.

As shown in the network representations of redistribution patterns in Figure 4, redistribution were not uniformly distributed across all machines. Instead, certain machines consistently absorbed increased workload during downtime events, suggesting asymmetric load‐balancing patterns within hospitals. Some of this asymmetry is due to the fact that the technical specifications are not matched across all machines. Within the hospital system studied, certain machines have specialized MLCs, energies, gantries, and couches (to name a few of the factors). As such, patients treated on certain machines could be transferred to others. For example, Machine 4 (Hospital C) has a high‐definition MLC and cannot generally absorb transfers. Also, Machine 1 (Hospital D) is an o‐ring gantry system and cannot absorb transfers. Nevertheless, our analysis showed measurable system‐level adaptation in response to machine interruptions.

**FIGURE 4:**
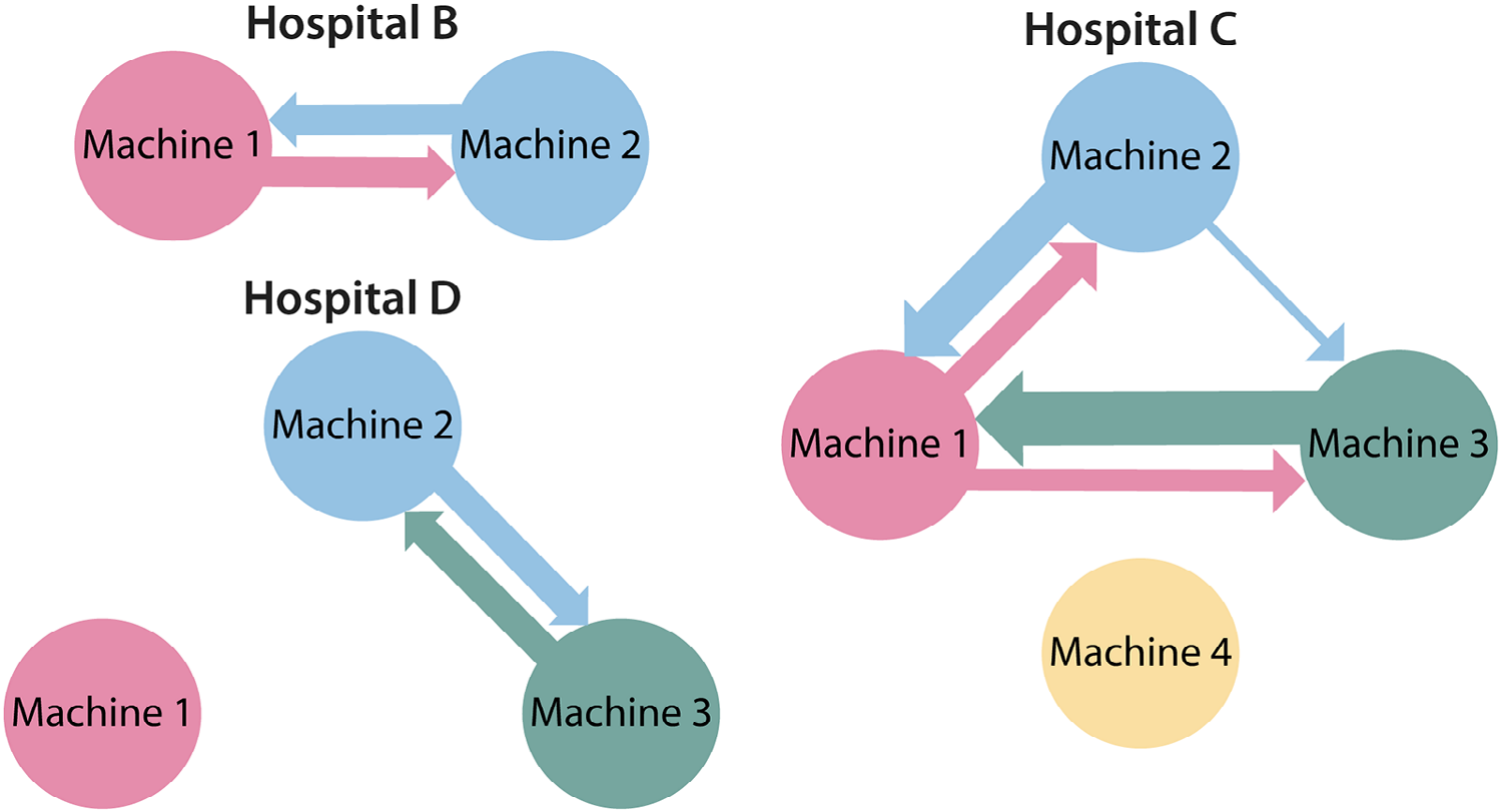
Network representation of intra‐hospital patient redistribution during linac downtime events across three hospitals. Nodes represent individual linear accelerators within each hospital. Arrow direction reflects the flow of redistributed patients, and arrow thickness is proportional to the magnitude of the mean increase in daily patient volume associated with downtime events.

### 3.4 Downtime tracking with a standardized reporting system

During the final 11 months of the 29‐month study period, the department placed increased emphasis on downtime reporting, supported by a standardized reporting form. During the initial 18‐month period of email‐based reporting, SPC achieved a sensitivity of 0.74, specificity of 0.99, PPV of 0.87, and NPV of 0.99. A subgroup analysis of this interval (Table 2) was conducted to assess whether the increased emphasis on reporting using a form affected SPC detection performance. Within this eleven‐month period, sensitivity was 0.64, specificity 0.99, positive predictive value 0.81, and negative predictive value 0.99. A Fisher's exact test comparing event reporting rates between the initial 18‐month and final 11‐month periods yielded a statistically significant result (p = 0.008), showing an increase in the number of events reported. A comparison of performance across all study subperiods is presented in Table 3.

**TABLE 2:** Performance of the SPC‐based downtime detection framework during the eleven‐month period following implementation of an improved structured downtime reporting process. Counts of true positive (manually‐reported and SPC‐predicted), false positive (SPC‐predicted without corresponding event report), and false negative (event log‐reported but not SPC‐predicted) events are shown by hospital and machine.

		True Positive (Manually Reported & SPC‐Predicted)	False Positive (SPC‐Predicted & Not Manually Reported)	False Negative (Manually Reported & Not Predicted by SPC)
**Hospital A**	Machine 1	2	2	0
**Hospital B**	Machine 1	5	2	5
	Machine 2	5	0	3
**Hospital C**	Machine 1	4	2	7
	Machine 2	4	0	0
	Machine 3	4	0	1
	Machine 4	4	0	1
**Hospital D**	Machine 1	4	1	0
	Machine 2	6	2	3
	Machine 3	5	1	4

**TABLE 3:** Summary of SPC‐based downtime detection performance across study subperiods. Sensitivity, specificity, PPV, and NPV are reported for the full 29‐month study period, the initial 18‐month period (email‐based reporting), the final 11‐month period following increased reporting emphasis, and the final 11‐month period with events less than one hour in duration excluded from the reference standard.

Data Length	Sensitivity	Specificity	PPV	NPV
29 Months	0.703	0.992	0.848	0.992
First 18 Months	0.739	0.992	0.867	0.992
Last 11 Months	0.642	0.993	0.811	0.993
*Last 11 Months* *(excluding < 1 hr events)*	0.768	0.996	0.811	0.996

Notably, the standardized reporting system captured a broader range of events than the prior email‐based process, including brief or clinically minor interruptions that therapists noted in comments (e.g., “down 15 min,” “treatment start delay 30 min”) but which did not result in measurable impacts on daily patient volume, operating hours, or cancellations. Excluding the 12 low‐impact events that were initially classified as false negatives, but are inherently undetectable by an operationally based SPC framework, performance improved during the eleven‐month study period to a sensitivity of 0.77, specificity of 1.00, PPV of 0.81, and NPV of 1.00. This subgroup analysis suggests that the decrease in sensitivity following adoption of a standardized reporting system reflects the expanded scope of event capture rather than a degradation in SPC detection performance.

## 4 DISCUSSION

### 4.1 SPC‐based detection of linac downtime

In this multi‐clinic evaluation of linac downtime across a single hospital system, SPC methods applied to routinely collected operational data identified downtime events with high specificity (0.99‐1.0) and negative predictive value (0.99‐1.0), indicating that normal operational days were rarely misclassified as downtime. In addition to detecting reported downtime events, SPC identified 23 events with operational signatures consistent with machine interruption that were not captured in the existing event reporting system. While some flagged days may have reflected true reductions in scheduled patient volume, several characteristics support their interpretation as downtime events, including abrupt deviations beyond established control limits and coincident increases in patient volume on alternate machines within the same clinic. Collectively, these findings suggest that traditional downtime reporting alone may underestimate the frequency of machine interruptions.

A key advantage of this framework is that it relies exclusively on data routinely collected in standard OIS enabling implementation with minimal additional infrastructure and can be automated through scripting. Automated monitoring of machine availability could support assessment of vendor uptime guarantees, benchmarking reliability across sites, evidence‐based capital planning, and equipment quality management programs. The enhanced reporting emphasis and structured reporting form introduced during the final eleven months of our study suggest that automated surveillance may help promote a stronger culture of downtime awareness and event documentation. During this period, agreement between SPC‐detected and reported events improved, with sensitivity increasing from 0.74 to 0.77.

Downtime events not attributable to systemic causes (i.e., IT or weather) comprised the majority of disruptions observed across the system. In contrast, weather‐ and IT‐related events occurred less frequently but were more likely to produce institution‐wide interruptions affecting multiple machines simultaneously. The predominance of mechanical and operational/uncharacterized events underscores the potential value of enhanced structured reporting: more granular documentation of failure modes could support identification of recurring machine‐specific issues, inform preventive maintenance scheduling, and reduce clinically disruptive interruptions over time.

A notable finding was the identification of compensatory patient redistribution during downtime events. Across the three hospitals with more than one linac, 41 machine interruptions were associated with increased activity on alternate linacs within the same institution, demonstrating a degree of operational resilience. Redistribution patterns were not uniform, likely reflecting differences in machine capabilities (i.e., high‐definition MLCs, o‐ring gantries), scheduling flexibility, and baseline utilization. Quantifying redistribution patterns may therefore provide insight into latent departmental capacity, identify operational bottlenecks, and support workforce and scheduling planning. Such data‐driven operational awareness represents a meaningful step toward proactive, rather than reactive, resource management in radiation oncology.

A period of increased departmental emphasis on downtime reporting, supported by a structured reporting form, resulted in significantly greater event documentation during the final eleven months of the study compared with the preceding 18 months (Fisher's exact test, p = 0.008). The expanded reporting captured not only operationally significant interruptions targeted by the SPC framework but also brief or minor events that produced no detectable changes in daily volume, cancellations, or operating hours likely due to increased vigilance by staff. Consequently, raw sensitivity during this period was lower than the overall 29‐month estimate (0.64 vs 0.70), but increased to 0.77 when analysis was restricted to events with measurable operational impact. Because some reported events lacked duration data and were conservatively classified as having measurable impact, this increase may be underestimated. These findings highlight the complementary nature of the two approaches: SPC identifies operationally significant disruptions, whereas structured reporting captures events below the detection threshold of metric‐based surveillance. The relative value of each method of downtime reporting depends on the department's operational goals. Accordingly, departments may benefit from a hybrid surveillance model that combines automated SPC‐based detection with structured downtime event reporting, leveraging the strengths of both approaches to improve downtime characterization, reduce reliance on ad hoc communication and improve institutional awareness of equipment reliability trends.

### 4.2 Methodological considerations for SPC‐based downtime detection

An important methodological question is whether a single metric, such as canceled appointments, is sufficient for downtime detection. Using cancellations alone reduced sensitivity from 0.70 to 0.47, indicating that many downtime events are better captured through concurrent reductions in patient volume and operating hours. Although PPV increased slightly from 0.79 to 0.81, this likely reflects the more conservative behavior of a single‐metric approach rather than improved precision. Collectively, these findings demonstrate that integrating multiple complementary operational metrics within an SPC framework improves sensitivity without sacrificing specificity. Institutions with access to reason‐coded cancellation data, such as Mosaiq classifications for machine‐down (M), planned break (B), patient no‐show (N), and general cancellation (X), may further improve specificity by restricting analyzes to machine‐related cancellations.

The moderate sensitivity (0.70) observed over the 29‐month study period reflects an inherent limitation of downtime detection using daily aggregate operational metrics. Brief interruptions or events occurring late in the treatment day often do not produce sufficient changes in patient volume, cancellations, or operating hours to exceed SPC control limits. This highlights a fundamental tradeoff between operational detectability and clinical impact, as events with minimal effect on daily throughput are difficult to identify using summary metrics alone.

Selection of the 45‐day rolling baseline window and ± 3σ control limits was guided by empirical evaluation across alternative parameter combinations. Rolling window lengths shorter than 30 days increased false positive rates, whereas windows longer than 60 days reduced sensitivity to sustained operational shifts. The 45‐day window provided a balance between responsiveness and baseline stability, and ± 3σ minimized overall classification error. Institutions adopting this framework may need to tune these parameters to reflect local scheduling patterns, machine mix, and reporting practices.

Finally, an important institutional consideration for the redistribution analysis is whether patients are routinely transferred between machines for scheduling reasons independent of downtime. At our institution, such transfers are uncommon outside of documented machine outages, and the > 4σ threshold applied here requires a large, abrupt single‐day volume increase that is unlikely to result from any gradual scheduling adjustments. However, in institutions where cross‐machine scheduling is more common, redistribution signals may reflect scheduling practices rather than downtime events, and detection parameters should be calibrated accordingly to the local workflows.

### 4.3 Comparison to literature

Recent work by Youn et al. demonstrated the use of vendor maintenance and service records, assisted by large language model classification, to characterize linac downtime patterns — an approach that captures hardware‐level failure detail and engineer response times not reflected in OIS scheduling data.^22^ Such approaches provide detailed information regarding technical failures, maintenance activities, and service response timelines, but do not directly quantify the clinical or operational consequences of machine interruptions. In contrast, the framework presented here operates directly on routinely collected OIS data and measures the operational footprint of downtime events, including reductions in patient volume, treatment cancellations, and redistribution of patients between machines. To our knowledge, no prior study has used SPC applied to operational radiotherapy data to automatically detect and quantify linac downtime. These approaches should therefore be viewed as complementary rather than competing. Integration of both data sources could support a more comprehensive assessment of machine reliability; however, multi‐institutional validation using prospectively collected, structured downtime records is needed to establish performance and generalizability across diverse clinical environments.

### 4.4 Limitations

Several limitations should be considered when interpreting these findings. First, this study was conducted within a single hospital system, which may limit generalizability to institutions with different staffing models, scheduling practices, or machine configurations. Nevertheless, the SPC framework is inherently adaptable because it relies on routinely collected operational metrics that are available in most commercial record‐and‐verify systems. Institutions implementing this approach may need to calibrate parameters such as baseline window length and control‐limit thresholds to reflect local workflows and machine utilization patterns. While the empirical parameter selection strategy described here provides a replicable approach for this calibration, prospective multi‐institutional validation is needed to establish performance and generalizable parameter ranges across diverse practice settings. Second, downtime event reports were used as the reference standard despite documented underreporting, likely resulting in conservative estimates of sensitivity and positive predictive value. The improved detection performance observed during the period of enhanced reporting suggests that true performance may be higher than reflected in our analysis. Finally, the use of daily aggregate operational metrics limits sensitivity for short‐duration interruptions that do not substantially affect daily throughput. Future work incorporating higher‐resolution operational data, such as appointment‐level timestamps and treatment delivery records, may improve detection of brief events and support real‐time monitoring capabilities.

## 5 CONCLUSION

Statistical process control applied to routinely collected treatment and scheduling data provides a practical and scalable framework for automated detection of linac downtime events. In this multi‐clinic study, SPC‐based monitoring demonstrated high specificity and identified additional events not captured through manual reporting alone. Downtime events were associated with measurable intra‐hospital redistribution of patient workload, highlighting the adaptive capacity of radiation oncology departments as well as potential operational stress points.

These findings support the integration of automated anomaly detection with structured downtime reporting to improve equipment surveillance, enhance operational transparency, and inform proactive resource planning in radiation oncology. Broader implementation and multi‐institutional validation may further establish the role of data‐driven operational monitoring in modern radiotherapy practice.

## AUTHOR CONTRIBUTIONS

Caroline E. Serafini and Hania Al‐Hallaq designed the methodology outlined in the paper. Caroline E. Serafini analyzed the data and made all figures in the manuscript. Matthew Thomas queried the internal systems to collect the machine data. Hania Al‐Hallaq and Matthew Thomas provided the reported downtime logs. Matthew Thomas, Xiaofeng Yang, Pretesh Patel, Justin Roper, Maxine Washington, Anees Dhabaan, and Hania Al‐Hallaq. provided insight into clinical operations and implementation of downtime tracking. Caroline E. Serafini and Hania Al‐Hallaq wrote and revised the paper. All authors reviewed the results and approved the final version of the paper.

## CONFLICT OF INTEREST STATEMENT

Hania Al‐Hallaq serves as a consultant for Varian Medical Systems as Chair of a consortium related to the Halcyon treatment platform.

## ETHICS STATEMENT

None. Statistical summaries used in this work were machine specific. No individual patient data was used. All experimental protocols were conducted under the guidelines of Emory University.

## Supporting information



Supporting Information

## Data Availability

Data and code underlying the results presented in this paper are not publicly available at this time. Materials used in the study are largely outlined in the Methods section; however, further details may be obtained from the corresponding author upon reasonable request.
