# Supplementary material for: Automated linac downtime tracking across 4 radiation oncology clinics within a single hospital system utilizing statistical process control
Source: J Appl Clin Med Phys. 2026 Oct 14;27(10):e70863. doi: 10.1002/acm2.70863 (PMC13669665; doi:10.1002/acm2.70863)
Supplement: Supplementary file 1 [file ACM2-27-e70863-s001.pdf]

Automated Linac Downtime Tracking Across 4 Radiation Oncology Clinics within a Single Hospital System Utilizing Statistical Process Control (Appendix)

Caroline E. Serafini[1], Matthew Thomas[1], Xiaofeng Yang[1], Pretesh Patel[1], Justin Roper[1], Maxine Washington[1], Anees Dhabaan[1], Hania Al-Hallaq[1]

[1]Department of Radiation Oncology and Winship Cancer Institute, Emory University, Atlanta, GA 30322, United States of America

Corresponding author: hania.al-hallaq@emory.edu

Appendix

S.A Determination of Sigma Number

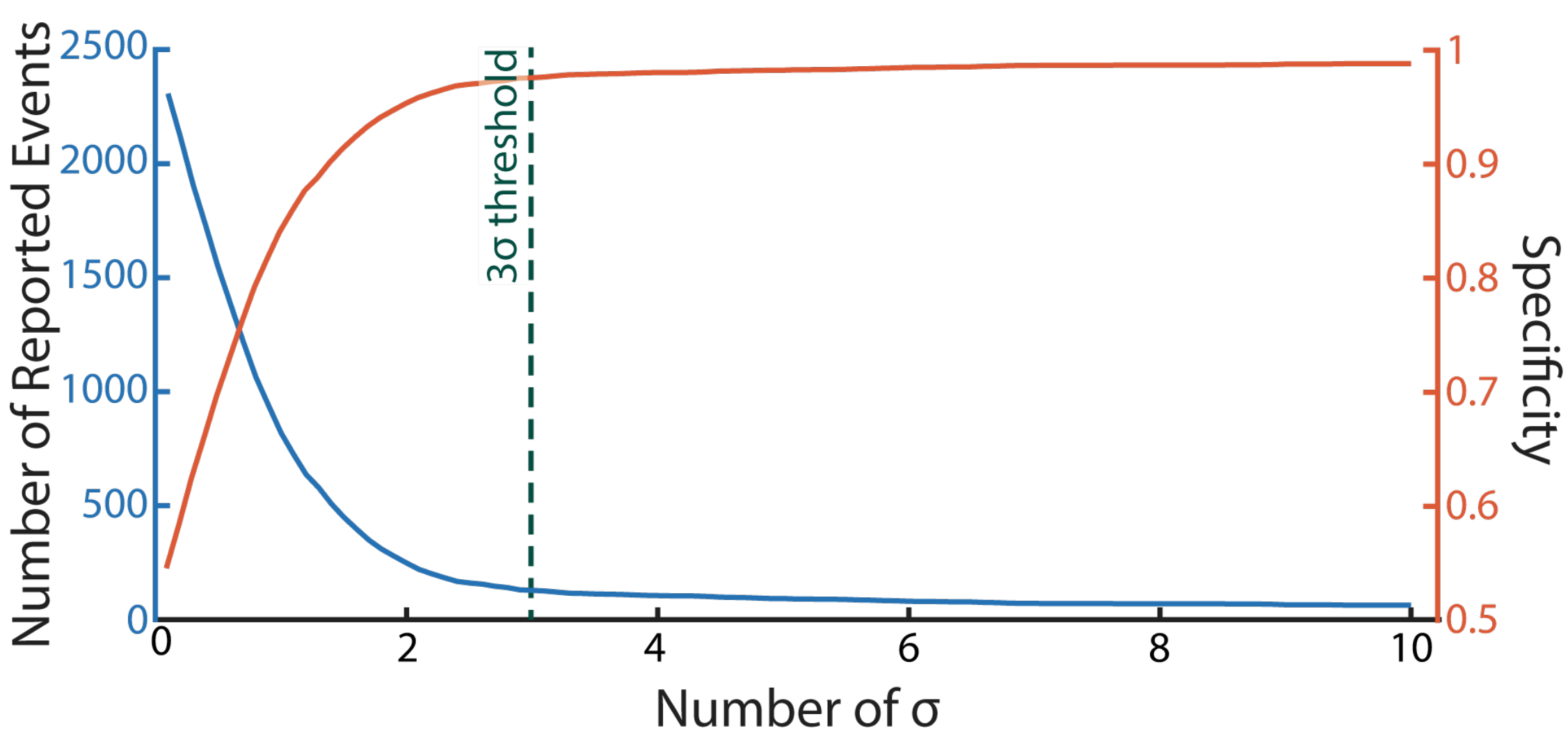

Figure S-1: Sensitivity analysis of control limit threshold selection for SPC-based downtime detection. The blue curve (left axis) shows the number of reported downtime events classified as anomalies as a function of the control limit threshold (σ), while the red curve (right axis) shows corresponding specificity. As the threshold increases, the number of flagged events decreases and specificity increases. A $\pm 3\sigma$ threshold (dashed line) provides a balance between minimizing false positives and maintaining detection sensitivity, consistent with conventional SPC practice for identifying special-cause variation.

S.B Determination of Rolling Window Size

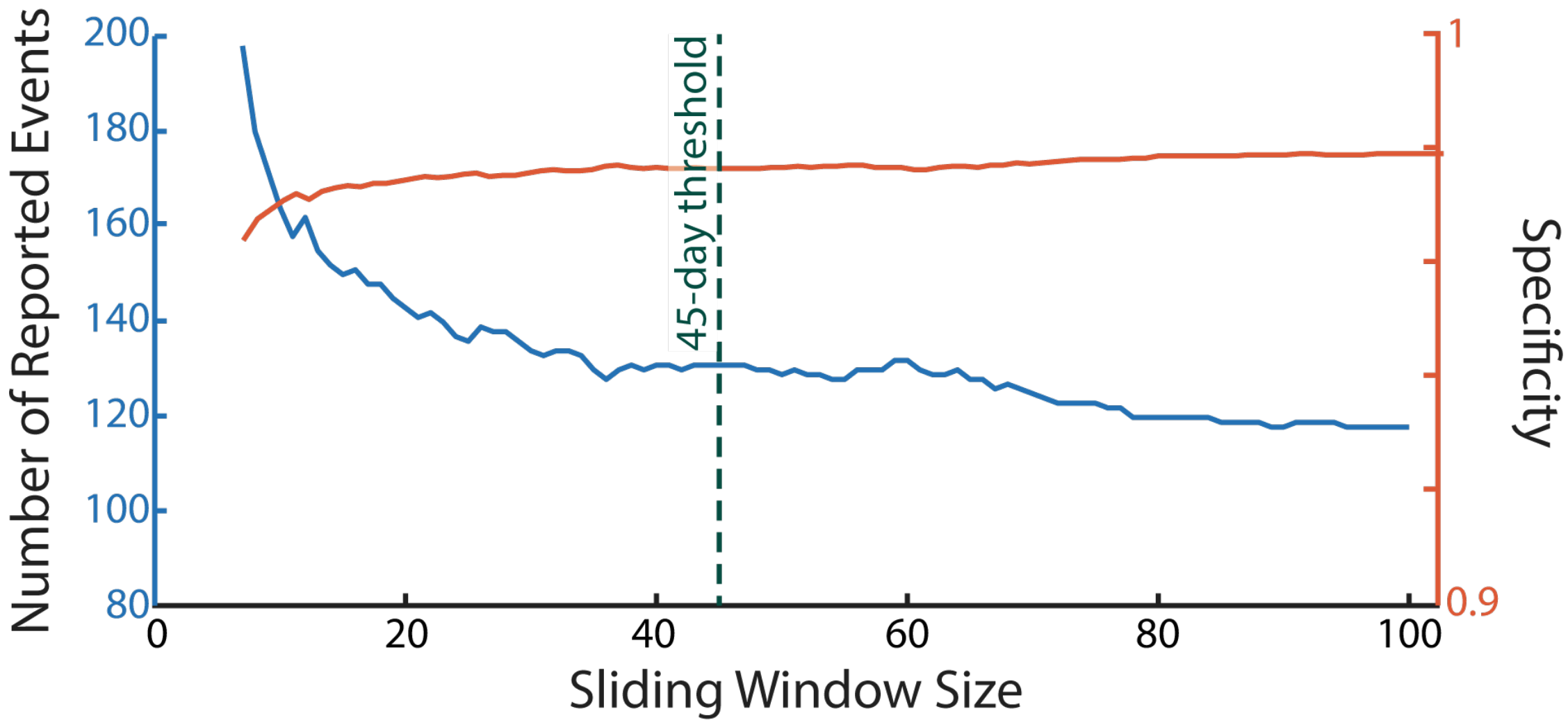

Figure S-2: Sensitivity analysis of rolling baseline window size used for SPC calculations. The blue curve (left axis) shows the number of reported downtime events classified as anomalies as a function of sliding window length, while the red curve (right axis) shows specificity. Shorter windows increase variability in baseline estimation, whereas longer windows reduce responsiveness to operational shifts. A 45-day rolling window (dashed line) provides a balance between baseline stability and sensitivity to meaningful deviations.

S.C SPC Charts: Patients / Day

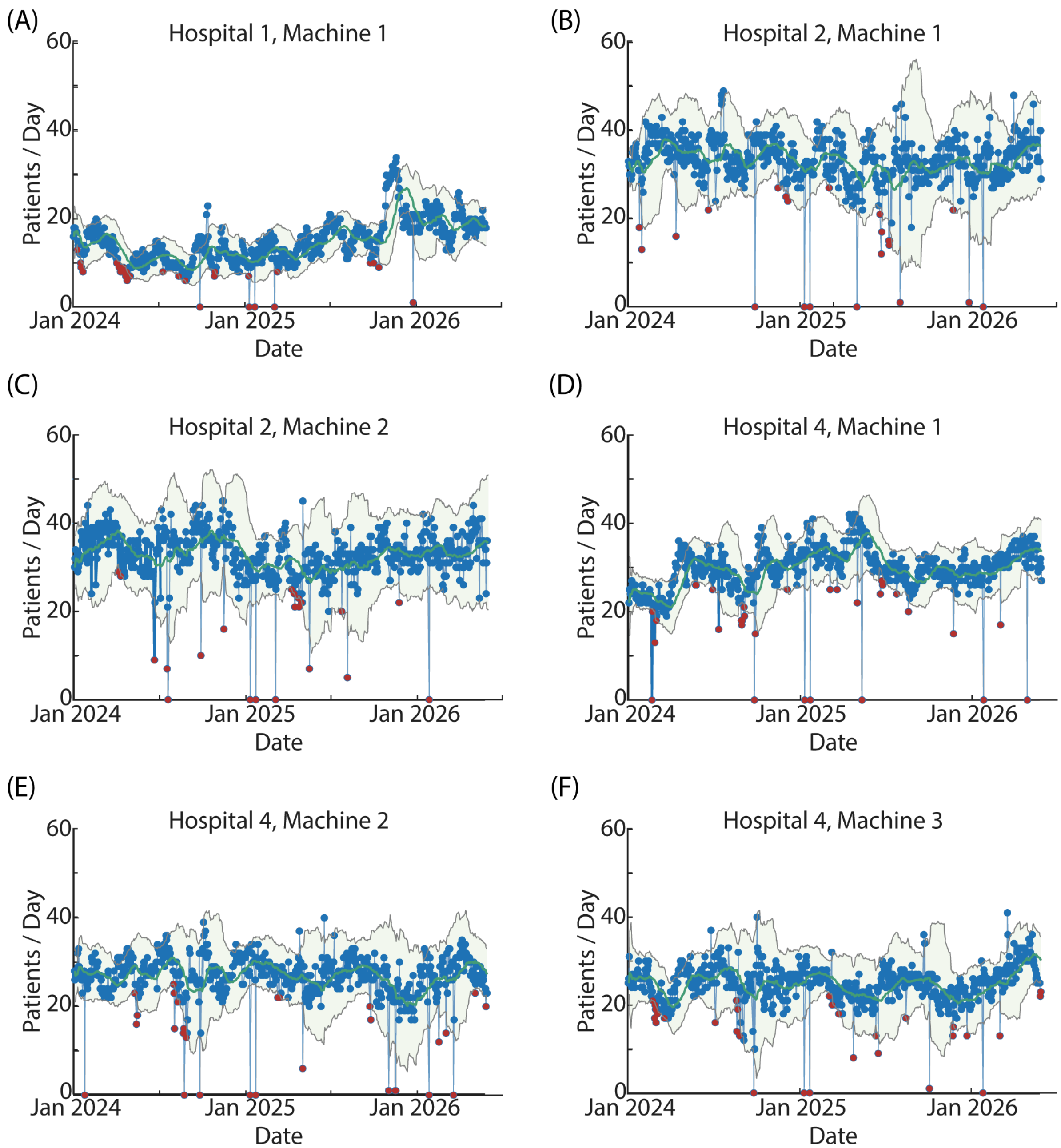

Figure S-3: Statistical process control (SPC) charts for the remaining six linacs over the 29-month study period. Daily patient volume is shown with rolling 45-day baseline means (solid line) and $\pm3\sigma$ control limits (shaded region). Points outside control limits represent special-cause variation. Red markers indicate log–reported downtime events, while additional SPC-flagged events without corresponding documentation are visible as control limit violations.

14 S.D SPC Charts: Operating Hours / Day

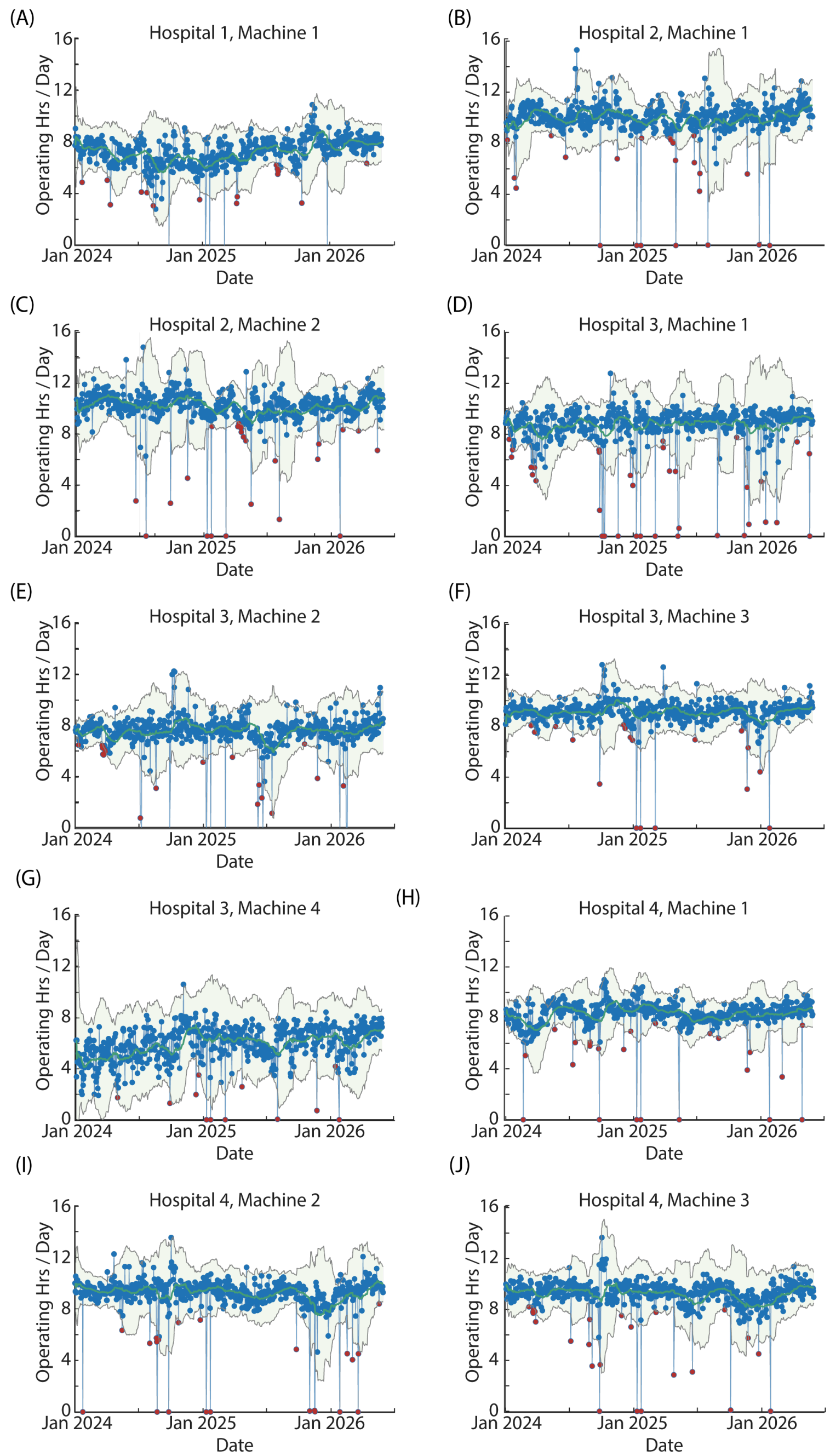

Figure S-4: Statistical process control (SPC) charts for 10 linacs over the 29-month study period. Daily operating hours are shown with rolling 45-day baseline means (solid line) and $\pm 3\sigma$ control limits (shaded region). Points outside control limits represent special-cause variation. Red markers indicate log–reported downtime events, while additional SPC-flagged events without corresponding documentation are visible as control limit violations.

S.E SPC Charts: Cancellations / Day

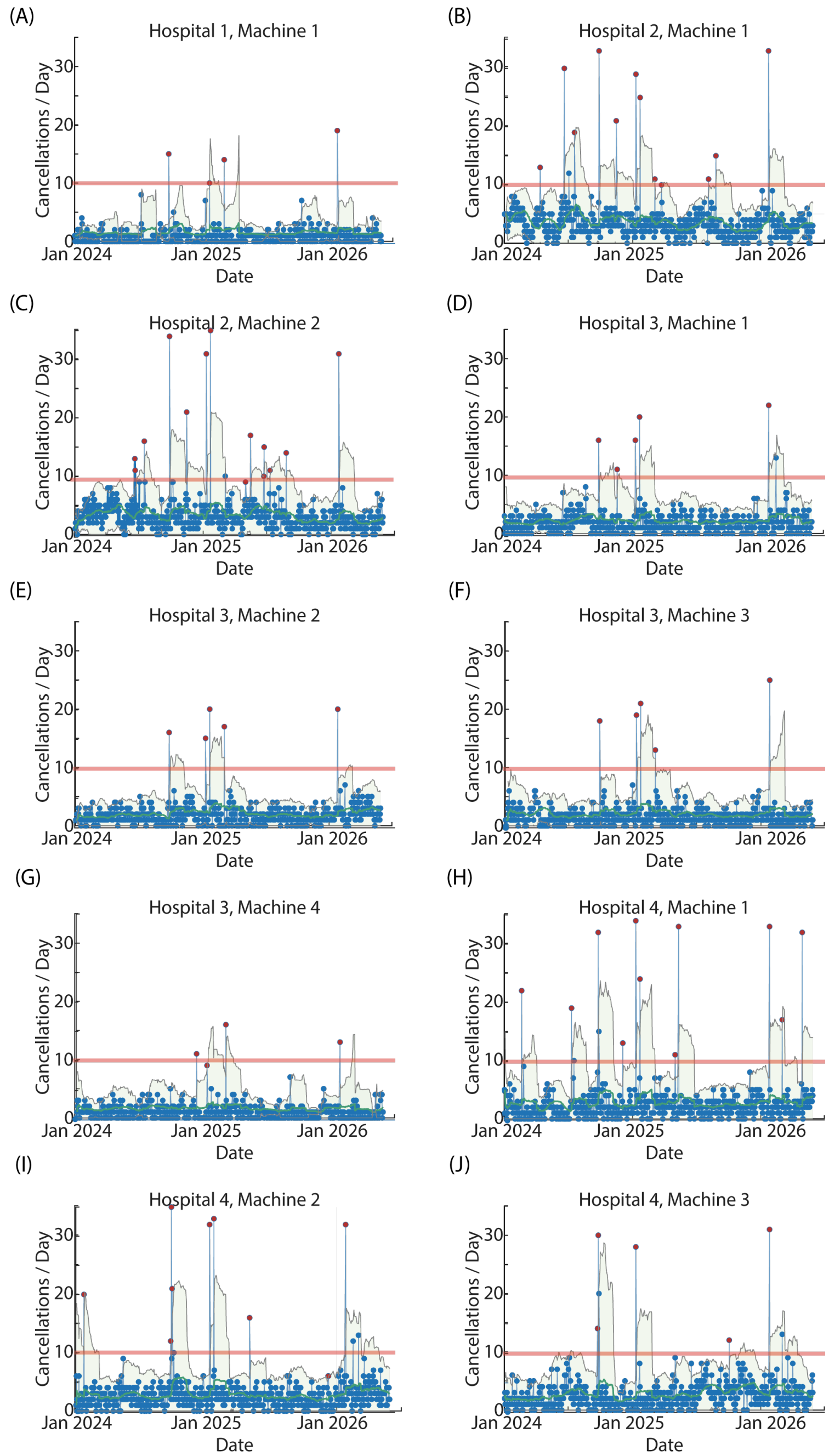

Figure S-5: Statistical process control (SPC) charts for 10 linacs over the 29-month study period. Daily number of cancelled patients are shown with rolling 45-day baseline means (solid line) and $\pm 3\sigma$ control limits (shaded region). Red line indicates 10 patient cancellations. Points outside control limits represent special-cause variation. Red markers indicate log–reported downtime events, while additional SPC-flagged events without corresponding documentation are visible as control limit violations.
